# Trained intensivist coverage and survival outcomes in critically ill patients: a nationwide cohort study in South Korea

**DOI:** 10.1186/s13613-023-01100-5

**Published:** 2023-01-13

**Authors:** Tak Kyu Oh, In-Ae Song

**Affiliations:** 1 Department of Anesthesiology and Pain Medicine, Seoul National University Bundang Hospital, Gumi-Ro, 173, Beon-Gil, Bundang-Gu, Seongnam, 13620 South Korea; 2 Department of Anesthesiology and Pain Medicine, College of Medicine, Seoul National University, Seoul, South Korea

**Keywords:** Cohort studies, Critical care, Intensive care unit, Mortality

## Abstract

**Background:**

The difference in survival outcomes between closed and open intensive care unit (ICU) designs with respect to trained intensivist coverage remains unknown. We aimed to investigate whether trained intensivist coverage is associated with mortality in critically ill patients admitted to the ICU in South Korea.

**Methods:**

This population-based cohort study used nationwide registration data from South Korea. This study enrolled all adult patients admitted to the ICU between January 1, 2016, and December 31, 2019. Patients, who were admitted ICU in a hospital that employed trained intensivists, were designated as the intensivist group.

**Results:**

This study included 1,147,493 critically ill patients admitted to the ICU. The intensivist and non-intensivist groups consisted of 484,004 (42.2%) and 663,489 (57.8%) patients, respectively. Mixed effect logistic regression revealed a 22% lower in-hospital mortality rate (odds ratio: 0.78. 95% confidence interval: 0.74, 0.81; *P* < 0.001) than that in the non-intensivist group. Mixed effect Cox regression revealed a 15% lower 1-year mortality rate (hazard ratio: 0.85. 95% confidence interval: 0.83, 0.89; *P* < 0.001) in the intensivist group than that in the non-intensivist group. Moreover, the in-hospital mortality was significantly lower in the intensivist group than that in the non-intensivist group, irrespective of age, Charlson comorbidity index, surgery or non-surgery associated admission, and invasive treatment during ICU stay.

**Conclusions:**

A closed ICU design with trained intensivist coverage was associated with lower in-hospital and 1-year mortality rates. Our results suggest that hospitals should employ trained intensivists to improve both short-term and long-term survival outcomes of critically ill patients.

**Supplementary Information:**

The online version contains supplementary material available at 10.1186/s13613-023-01100-5.

## Background

The intensive care unit (ICU) is designed for the management of critically ill patients who require greater support and attention than is available in the general ward [1]. The first ICU was established in the late 1950s, and critical care medicine has improved since then [2, 3]. Currently, the ICU plays a critical role in monitoring critically ill patients and providing intervention and organ support [4].

The physician staffing pattern in the ICU is an important issue in critical care [5]. Previous studies have reported decreased mortality in patients treated in a closed ICU, where they were admitted under the full responsibility of a trained intensivist [5–8]. This is because invasive procedures or decisions regarding life-sustaining therapies in ICU patients might be influenced by trained intensivists [9]. However, a recent meta-analysis of 90 studies with 444,042 patients concluded that total mortality did not differ between the closed type of ICU with trained intensivist coverage and the open type of ICU without trained intensivist coverage [10]. Thus, the difference in survival outcomes between closed and open ICU designs according to trained intensivist coverage remains unknown. The South Korean government has implemented a special payment system for closed design ICUs with intensivist coverage since August 2015 by law. The impact of intensivist coverage on the survival outcomes of critically ill patients should be evaluated using a nationwide registration database.

Therefore, we aimed to investigate the manner in which trained intensivist coverage was associated with mortality among critically ill patients admitted to the ICU in South Korea.

## Methods

### Study design, setting, and ethical concerns

This population-based retrospective cohort study was conducted in accordance with the guidelines of Strengthening the Reporting of Observational Studies in Epidemiology [11]. The Institutional Review Board (IRB) of Seoul National University Bundang Hospital exempted deliberation of the study protocol, because we used public data open to all researchers (IRB number: X-2102–666-904). The IRB also waived the requirement of informed consent, because data analysis was performed retrospectively in an anonymized form.

### Database

The national health insurance service (NHIS) database of South Korea was used as the data source. As the NHIS is the sole public health insurance system in South Korea, it contains all data on disease diagnoses according to the International Diseases and Related Health Issues 10th edition (ICD-10) codes and prescription information on all drugs and/or procedures. The NHIS permitted data sharing after approving the study protocol (NHIS-2021–1-620).

### Study population

We included all adult patients (≥ 20 years) who were admitted to the ICU from 2016 to 2019 in South Korea. The prescription code of ICU admission during hospitalization was used for data extraction; no patient was admitted to the ICU in South Korea without registering the prescription code. If a patient was admitted to the ICU twice or more during the study period, only the last ICU admission on the latest date was included in this study, because mortality after ICU admission was one of the endpoints of our study. Patients with missing data on important demographic variables (age and sex) were excluded from the analysis.

### Intensivist coverage ICU in South Korea

The South Korean government established a special payment system by law only for hospitals that employed trained intensivists for ICU staffing. This payment system is implemented for hospitals to employ an intensivist for ICU staffing under the condition that the intensivist should work only in the ICU for ≥ 8 h/day and ≥ 5 days/week. Moreover, the law requires that there should be at least one trained intensivist per ICU. The intensivist has to be a certified specialist physician from the Korean Society of Critical Care Medicine after specific fellowship training in critical care medicine. Doctors specializing in internal medicine, anesthesiology and pain medicine, pediatrics, neurology, neurosurgery, emergency medicine, general surgery, and thoracic surgery can apply for the fellowship training course for trained intensivists, which lasts for 1 year. In 2022, there were 1774 trained intensivists in South Korea, comprising 316 (18%) anesthesiologists, 546 (30%) doctors of internal medicine, 201 (12%) neurosurgeons, 196 (11%) doctors of emergency medicine, 196 (11%) thoracic surgeons, 129 (7%) general surgeons, 118 (7%) doctors of neurology, and 72 (4%) pediatricians. As this special payment system for intensivist coverage in South Korea was initiated in August 2015, our study commenced on January 1, 2016. Patients who were admitted to the ICU in a hospital that employed trained intensivists were designated as the intensivist group, whereas the other patients were denoted the non-intensivist group.

### Study endpoints

The primary endpoint was in-hospital mortality after ICU admission. The secondary endpoint was the 1-year all-cause mortality after ICU admission, defined as any death within 1 year after the date of ICU admission.

### Study parameters

Age and sex were collected as demographic variables. Socioeconomic status-related information, such as the employment status, national household income level, and residence at ICU admission, were collected. Residence was classified into two groups: urban (Seoul and other metropolitan cities) and rural (all other areas). The NHIS contains data on patients’ household income level to determine insurance premiums for the year, and approximately 67% of medical expenses are subsidized by the government [12]. However, the Medical Aid Program includes individuals who cannot afford insurance premiums or have difficulty supporting themselves financially. In this program, the government covers nearly all medical expenses to minimize the financial burden. All patients were divided into five groups using the quartile ratio, in addition to the Medical Aid Program group. The length of hospital stay (LOS) (days) and ICU stay were collected. The admitting departments were classified into two groups [internal department (IM) or non-IM]. We determined whether or not patients were admitted to the hospital through the emergency room (ER). If patients underwent surgery during hospitalization, it was considered a surgery-associated hospital admission. Data regarding whether the patient was admitted to the isolated ICU were collected. The Charlson comorbidity index (CCI) was calculated using ICD-10 codes according to previous study, to reflect the patient’s comorbid disease status [13]. Patients were classified into three groups according to the level of the hospital to whose ICU they were admitted. The results of hospitalization were classified into four groups as follows: 1) same hospital follow-up, 2) transfer to a long-term care center facility, 3) death during hospitalization, and 4) discharge and other outpatient clinic follow-ups. The date of death during hospitalization or hospital discharge was also collected. The total cost for hospitalization was recorded in United States Dollars (USD).

### Statistical analysis

The clinicopathological characteristics of patients were presented as the mean with standard deviation (SD) for continuous variables and numbers with percentages for categorical variables. The clinicopathological characteristics of the intensivist and non-intensivist groups were compared using the *t* test and chi-squared test for continuous and categorical variables, respectively. A hierarchical approach was used to account for clustering of the covariates at the level of the hospital, where patients were admitted to the ICU. For hierarchical cluster analysis, agglomerative clustering was performed using hospital-related variables, including hospital location, total number of doctors, type of hospital (general tertiary hospital, general hospital, and other hospitals), specialist doctors, nurses, and pharmacists, total number of hospital beds, and total number of operating rooms. Three groups were created based on the results of hierarchical clustering analysis; the characteristics of the three hospital groups are presented in Additional file 1: Table S1.

Thereafter, as the hospital level variable could be interdependent with patient-related variables as clusters, we constructed a mixed effect logistic regression model for in-hospital mortality in patients admitted to the ICU. All covariates were included in the mixed effect logistic regression model for adjustment, and the results were presented as odds ratios (ORs) with 95% confidence intervals (CIs), considering the random effect of the hospital level.

We also constructed a mixed effects Cox regression model for 1-year mortality in patients admitted to the ICU considering the random effect of the hospital level. The results were presented as hazard ratios (HRs) with 95% CIs. Moreover, we performed subgroup analyses according to age (> 65 or ≤ 65 years), CCI (> 3 or ≤ 3), surgery or non-surgery associated admission, and invasive treatment during ICU stay, such as mechanical ventilation, continuous renal replacement therapy (CRRT), and extracorporeal membrane oxygenation (ECMO) support. There was no issue regarding multi-collinearity between variables in all models with the criterion of variance inflation factors < 2.0. All statistical analyses were performed using R software (version 4.0.3; R Foundation for Statistical Computing, Vienna, Austria), and statistical significance was set at *P* < 0.05.

## Results

### Study population

**Fig. 1:**
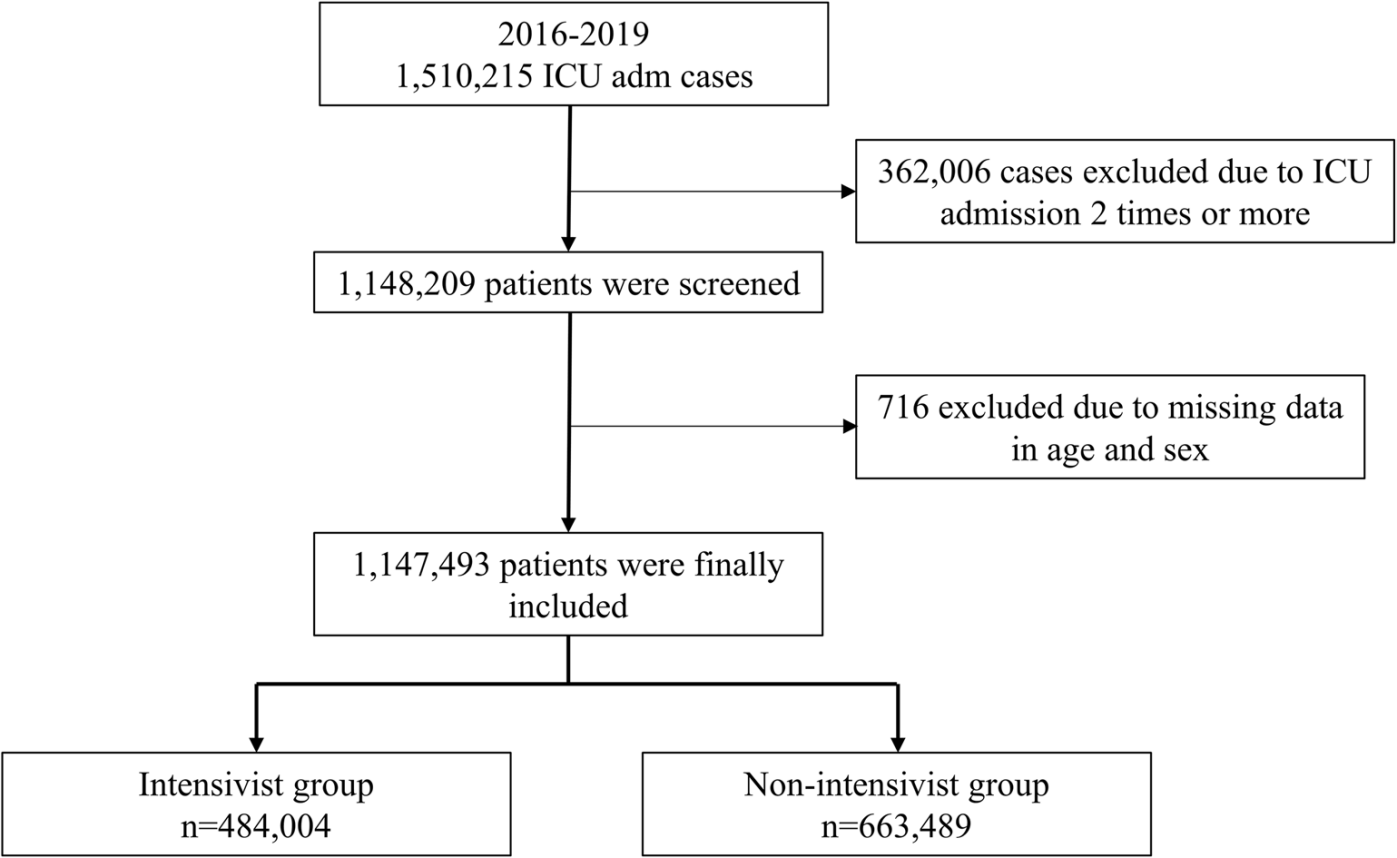
Flowchart depicting the patient selection process

A total of 1,510,215 ICU admissions were performed in South Korea between January 1, 2019, and December 31, 2019. A total of 362,006 cases of patients with ≥ 2 ICU admissions during the study period were excluded, to restrict focus on only the last ICU admission on the latest date. We also excluded 716 patients whose data on age and sex were missing. Finally, 1,147,493 critically ill patients who were admitted to ICU were included in this study, of which 484,004 (42.2%) patients were admitted to an ICU with trained intensivist coverage (intensivist group), while 663,489 (57.8%) patients were admitted to an ICU without trained intensivist coverage (non-intensivist group) (Fig. 1). Additional file 2: Table S2 shows the clinicopathological characteristics of all patients included in this study. The mean age was 68.4 years (SD: 15.3 years), and the proportion of men was 57.2% (657,331/1,147,493). The mean duration of ICU stay and hospitalization was 3.6 days (SD: 5.4 days) and 14.3 days (12.5 days), respectively. Surgery accounted for 69.0% (792,245/1,147,493) of ICU admissions. The 30-day, 90-day, and 1-year mortality rates after ICU admission were 17.2% (196,835/1,147,493), 22.8% (262,161/1,147,493), and 29.8% (341,414/1,147,493), respectively.

Table 1 depicts the comparison of the clinicopathological characteristics between the intensivist and non-intensivist groups. The 30-day, 90-day, and 1-year mortality rates in the intensivist group were 16.0%, 21.6%, and 28.3%, respectively, which were significantly lower than those in the non-intensivist group at 18.0%, 23.8%, and 30.8% (*P* < 0.001), respectively. The mean total cost for hospitalization in the intensivist group was 11,129.9 USD (10,787.2 USD), which was higher than that in the non-intensivist group at 6,766.4 USD (6,839.0 USD) (*P* < 0.001).

**Table 1:** Comparison of clinicopathological characteristics between the intensivist and non-intensivist groups

Variable	Intensivist group *n* = 484,004	Non-intensivist group *n* = 663,489	*P* value
Age, year	66.4 (15.1)	69.9 (15.2)	< 0.001
Sex, men	284,708 (58.8)	371,623 (56.0)	< 0.001
Having a job	261,814 (54.1)	335,814 (54.1)	< 0.001
Household income level			< 0.001
Medical aid program	35,737 (7.4)	74,683 (11.3)	
Q1 (Lowest)	77,556 (16.0)	109,691 (16.5)	
Q2	73,500 (15.2)	95,803 (14.4)	
Q3	947,648 (19.6)	123,721 (18.6)	
Q4 (Highest)	151,242 (31.2)	185,700 (28.0)	
Unknown	51,321 (10.6)	73,891 (11.1)	
Residence			< 0.001
Urban area	209,851 (43.4)	204,446 (30.8)	
Rural area	228,719 (47.3)	393,635 (59.3)	
Unknown	45,434 (9.4)	65,408 (9.9)	
ICU stay, day	3.5 (5.1)	3.8 (5.6)	< 0.001
LOS, day	14.9 (12.6)	13.8 (12.4)	< 0.001
CCI, point	2.5 (2.2)	2.5 (2.1)	< 0.001
Admitting department			< 0.001
Non-IM	250,575 (51.8)	312,457 (47.1)	
IM	233,429 (48.2)	351,032 (52.9)	
Hospital admission through ED	296,553 (61.3)	367,726 (55.4)	< 0.001
Isolated ICU admission	18,579 (3.8)	13,559 (2.0)	< 0.001
Hospital level^a^			< 0.001
Tertiary general hospital	327,440 (67.7)	147,310 (22.2)	
General hospital	156,564 (32.3)	499,383 (75.3)	
Other hospital	0 (0.0)	16,796 (2.5)	
Surgery associated hospital admission	384,845 (79.5)	407,400 (61.4)	< 0.001
Result of hospitalization			< 0.001
Same-hospital follow-up	87,933 (18.2)	122,117 (18.4)	
Transfer to a long-term facility care center	27,730 (5.7)	23,219 (3.5)	
Death during hospitalization	71,178 (14.7)	101,623 (15.3)	
Discharge and other outpatient clinic follow-up	297,163 (61.4)	416,530 (62.8)	
30 d mortality	77,627 (16.0)	119,208 (18.0)	< 0.001
90 d mortality	104,423 (21.6)	157,738 (23.8)	< 0.001
1-year mortality	137,046 (28.3)	204,368 (30.8)	< 0.001
Total cost for hospitalization, USD	11,129.9 (10,787.2)	6,766.4 (6,839.0)	< 0.001
Year of admission			< 0.001
2016	88,005 (18.2)	172,465 (26.0)	
2017	116,155 (24.0)	158,587 (23.9)	
2018	130,782 (27.0)	155,802 (23.5)	
2019	149,062 (30.8)	176,635 (26.6)	

*ICU* intensive care unit; *LOS* length of hospital stays; *CCI* Charlson comorbidity index; *IM* internal medicine; *ED* emergency department; *ECMO* extracorporeal membrane oxygenation; *CRRT* continuous renal replacement therapy; *USD* United States Dollars

^a^Hospital location, type of hospital, total number of doctors, specialist doctors, nurses, and pharmacists, total number of hospital beds, and total number of operating rooms were used for hierarchical approach to account for clustering at the level of the hospital. Detailed information was presented in Additional file 2: Table S2

### Survival analysis

Table 2 shows the results of the mixed effect logistic regression model for in-hospital mortality after ICU admission. The in-hospital mortality rate was 22% lower in the intensivist group (OR: 0.78. 95% CI 0.74, 0.81; *P* < 0.001) than that in the non-intensivist group. Table 3 depicts the results of mixed effect Cox regression model for 1-year mortality after ICU admission. The 1-year mortality rate was 15% lower in the intensivist group (HR: 0.85. 95% CI 0.83, 0.89; *P* < 0.001) than that in the non-intensivist group.

**Table 2:** Mixed effect logistic regression model for in-hospital mortality

Variable	OR (95% CI)	*P* value
Intensivist group (non-intensivist group)	0.78 (0.74, 0.81)	< 0.001
Age, year	1.02 (1.02, 1.03)	< 0.001
Sex, men (vs women)	1.10 (1.08, 1.12)	< 0.001
Having a job (vs unemployment)	0.96 (0.93, 0.97)	< 0.001
Household income level		
Q1 (Lowest) (vs medical aid program)	0.83 (0.80, 0.85)	< 0.001
Q2 (vs medical aid program)	0.82 (0.79, 0.85)	< 0.001
Q3 (vs medical aid program)	0.82 (0.79, 0.83)	< 0.001
Q4 (Highest) (vs medical aid program)	0.83 (0.80, 0.85)	< 0.001
Unknown (vs medical aid program)	0.85 (0.79, 0.89)	< 0.001
Residence		
Rural area (vs Urban area)	1.02 (1.00, 1.04)	0.087
Unknown (vs Urban area)	1.55 (1.47, 1.65)	< 0.001
CCI, point	1.11 (1.11, 1.12)	< 0.001
Admitting department		
IM (vs non-IM)	2.05 (2.03, 2.10)	< 0.001
Hospital admission through ER	1.30 (1.28, 1.33)	< 0.001
Hospital level^a^		
B (vs A)	1.48 (1.35, 1.52)	< 0.001
C (vs A)	0.59 (0.57, 0.60)	< 0.001
Isolated ICU admission	1.63 (1.58, 1.69)	< 0.001
Surgery associated hospital admission	0.75 (0.73, 0.77)	< 0.001
Year of admission		
2017 (vs 2016)	0.90 (0.88, 0.92)	< 0.001
2018 (vs 2016)	0.89 (0.87, 0.90)	< 0.001
2019 (vs 2016)	0.75 (0.73, 0.77)	< 0.001

*OR* odds ratio; *CI* confidence interval; *ICU* intensive care unit; *CCI* Charlson comorbidity index; *IM* internal medicine; *ED* emergency department; *ECMO* extracorporeal membrane oxygenation; *CRRT* continuous renal replacement therapy

^a^Hospital location, type of hospital, total number of doctors, specialist doctors, nurses, and pharmacists, total number of hospital beds, and total number of operating rooms were used for hierarchical approach to account for clustering at the level of the hospital. Detailed information was presented in Additional file 1: Table S1

**Table 3:** Mixed effects Cox regression model for 1-year mortality

Variable	HR (95% CI)	*P* value
Intensivist group (non-intensivist group)	0.85 (0.83, 0.89)	< 0.001
Age, year	1.03 (1.03, 1.04)	< 0.001
Sex, men (vs women)	1.18 (1.17, 1.19)	< 0.001
Having a job (vs unemployment)	0.96 (0.94, 0.97)	< 0.001
Household income level		
Q1 (Lowest) (vs medical aid program)	0.84 (0.82, 0.86)	< 0.001
Q2 (vs medical aid program)	0.84 (0.83, 0.85)	< 0.001
Q3 (vs medical aid program)	0.80 (0.78, 0.82)	< 0.001
Q4 (Highest) (vs medical aid program)	0.78 (0.76, 0.80)	< 0.001
Unknown (vs medical aid program)	0.88 (0.85, 0.90)	< 0.001
Residence		
Rural area (vs Urban area)	1.04 (1.03, 1.05)	< 0.001
Unknown (vs Urban area)	1.52 (0.47, 1.56)	< 0.001
CCI, point	1.12 (1.11, 1.13)	< 0.001
Admitting department		
IM (vs non-IM)	1.67 (1.64, 1.69)	< 0.001
Hospital admission through ER	1.34 (1.33, 1.35)	< 0.001
Hospital level^a^		
B (vs A)	1.32 (1.31, 1.33)	< 0.001
C (vs A)	0.70 (0.68, 0.73)	< 0.001
Isolated ICU admission	1.30 (1.28, 1.32)	< 0.001
Surgery associated hospital admission	0.76 (0.76, 0.77)	< 0.001
Year of admission		
2017 (vs 2016)	0.96 (0.95, 0.97)	< 0.001
2018 (vs 2016)	0.85 (0.84, 0.86)	< 0.001
2019 (vs 2016)	0.84 (0.83, 0.85)	< 0.001

*HR* hazard ratio; *CI* confidence interval; *ICU* intensive care unit; *CCI* Charlson comorbidity index; *IM* internal medicine; *ED* emergency department; *ECMO* extracorporeal membrane oxygenation; *CRRT* continuous renal replacement therapy

^a^Hospital location, type of hospital, total number of doctors, specialist doctors, nurses, and pharmacists, total number of hospital beds, and total number of operating rooms were used for hierarchical approach to account for clustering at the level of the hospital. Detailed information was presented in Additional file 1: Table S1

### Sensitivity and subgroup analyses

Table 4 presents the results of the subgroup analyses. Subgroup analysis revealed that the in-hospital mortality was significantly lower in the intensivist group than that in the non-intensivist group, irrespective of age (> 65 or ≤ 65 years), CCI (> 3 or ≤ 3 points), surgery or non-surgery associated admission, and invasive treatment during ICU stay, such as mechanical ventilation, CRRT, or ECMO support.

**Table 4:** Subgroup analyses for in-hospital mortality

Variable	OR (95% CI)	*P* value
Age > 65 years (*n* = 689,525)		
Intensivist group (non-intensivist group)	0.70 (0.68, 0.73)	< 0.001
Age ≤ 65 years (*n* = 457,968)		
Intensivist group (non-intensivist group)	0.75 (0.73, 0.77)	< 0.001
CCI ≤ 3 (*n* = 860,894)		
Intensivist group (non-intensivist group)	0.70 (0.68, 0.72)	< 0.001
CCI > 3 (*n* = 286,599)		
Intensivist group (non-intensivist group)	0.78 (0.76, 0.79)	< 0.001
Surgery associated admission		
Intensivist group (non-intensivist group)	0.77 (0.75, 0.78)	< 0.001
Non-surgery associated admission		
Intensivist group (non-intensivist group)	0.70 (0.68, 0.72)	< 0.001
Mechanical ventilator support group		
Intensivist group (non-intensivist group)	0.73 (0.71, 0.75)	< 0.001
No mechanical ventilator support group		
Intensivist group (non-intensivist group)	0.78 (0.77, 0.81)	< 0.001
CRRT use group		
Intensivist group (non-intensivist group)	0.95 (0.91, 0.98)	0.047
No CRRT group		
Intensivist group (non-intensivist group)	0.70 (0.68, 0.71)	< 0.001
ECMO support group		
Intensivist group (non-intensivist group)	0.92 (0.78, 0.97)	0.046
No ECMO support group		
Intensivist group (non-intensivist group)	0.73 (0.71, 0.74)	< 0.001

*OR* odds ratio; *CI* confidence interval; *ICU* intensive care unit; *CCI* Charlson comorbidity index; *CRRT* continuous renal replacement therapy; *ECMO* extracorporeal membrane oxygenation

## Discussion

This population-based cohort study found that a closed-type ICU with trained intensivist coverage was associated with lower in-hospital and 1-year mortality after ICU admission in South Korea, irrespective of age, CCI, surgery or non-surgery associated admission, hospital level, and invasive treatment during ICU stay. We showed that a closed ICU design with trained intensivist coverage may improve survival outcomes among critically ill patients in the ICU using nationwide registration big data.

Trained intensivists play many important roles in modern ICUs. They usually assess the patient in the ward during consultation, give expert advice, and make quick decisions [14]. Intensivists also determine admission of critically ill patients to the ICU and make final treatment decisions during ICU stay as leaders of the ICU team [14]. Moreover, the intensivist can determine the decision not-to-treat and end-of-life care for terminally ill patients [14, 15]. However, according to a global ICU needs assessment survey in 2020, 44% of ICUs were of the open type without intensivist coverage in 34 countries [16]. Although the South Korean government implements a special payment system for hospitals that employ trained intensivists, only 41.1% of critically ill patients could receive ICU care in hospitals that employed trained intensivists in South Korea.

Previous studies have reported the impact of intensivist coverage on the clinical outcomes of critically ill patients [5–8, 10]. Vahedian-Azimi et al. performed the most recent meta-analysis in 2021 [10]. They analyzed 444,042 patients from 90 studies and reported the superiority of closed vs open ICUs with respect to the in-hospital and ICU mortality rates and ICU LOS, with no difference in total mortality or severity of illness [10]. The current study also showed that the closed type of ICU with intensivist coverage was associated with lower in-hospital mortality using a larger sample size (*n* = 1,147,493). Moreover, we showed that long-term mortality up to 1 year after ICU admission was also associated with intensivist coverage. This finding is valuable, because the factors associated with long-term prognosis in ICU survivors post-ICU discharge constitute emerging issues in critical care medicine [17]. Therefore, the relationship between the closed ICU model with intensivist coverage and long-term prognosis among survivors of critical illness warrants further investigation.

The findings of the subgroup analyses are important. Patients requiring invasive treatment such as mechanical ventilatory support, CRRT, or ECMO support may constitute a high-risk population (with organ failure) for increased in-hospital mortality during ICU stay. Therefore, we postulated that the impact of intensivist coverage on the survival outcomes of patients who underwent invasive treatment would be more significant. However, the OR for in-hospital mortality was higher in the CRRT group and ECMO group than that in the non-CRRT and non-ECMO groups. This result should be interpreted cautiously, because recent guidelines of the Society of Critical Care Medicine and the Extracorporeal Life Support Organization recommend that ECMO is best performed by a multidisciplinary team, which intensivists are positioned to engage and lead [18]. A recent retrospective cohort study reported that an intensivist-led multidisciplinary team was associated with improved clinical outcomes in patients who underwent venovenous ECMO support (19). Various factors could have affected this result. For example, intensivists may select the indication of ECMO or CRRT more widely than non-intensivists, which would affect the results. Thus, the impact of a closed ICU with intensivist coverage should be studied further for patients who undergo ECMO support, CRRT use, or mechanical ventilatory support.

Our study has several limitations. First, we could not distinguish hospitals according to specific intensivist coverage patterns, such as 24/7 coverage. There may be differences in the outcomes of ICU patients according to 24/7 or daytime coverage by the intensivist. Second, we did not adjust for factors, such as body mass index, exercise, alcohol consumption, and smoking, because of the lack of information from the NHIS database. Third, although the contribution of intensivist coverage to the excellent survival outcome in critically ill patients is highly likely, it is difficult to determine whether it is the sole factor responsible for this effect. For example, trained nurses and the quality of hospital systems may affect the survival outcomes of critically ill patients, in addition to intensivist coverage. Fourth, we did not examine whether there was a dose relationship between the number of intensivists and survival outcomes after ICU admission, because we could not accurately assess the number of intensivists in all hospitals in South Korea. Finally, there might be a bias arising from patients who were not admitted to the ICU, because they were too sick or the number of ICU beds was insufficient.

## Conclusion

This population-based cohort study showed that a closed ICU design with trained intensivist coverage was associated with lower in-hospital and 1-year mortality rates, irrespective of age, CCI, surgery or non-surgery associated admission, hospital level, and invasive treatment during ICU stay. Our result suggested that hospitals should employ trained intensivists to improve the short-term and long-term survival outcomes of critically ill patients.

## Supplementary Information

**Additional file 1: Table S1**. Characteristics of three hospital groups (total 369 hospitals).

**Additional file 2: Table S2**. Clinicopathological characteristics of all patients (n=1,147,493).

## Data Availability

All data are available upon reasonable request from the corresponding author.

## Abbreviations

ICU: Intensive care unit
NHIS: National health insurance service
ICD-10: International Classification of Diseases External 10th Revision
CCI: Charlson comorbidity index
IRB: Institutional Review Board
IM: Internal medicine
LOS: Length of hospital stay
ER: Emergency room
ECMO: Extracorporeal membrane oxygenation
CRRT: Continuous renal replacement therapy
SD: Standard deviation
OR: Odds ratio
CI: Confidence interval
