# Supplementary material for: Trained intensivist coverage and survival outcomes in critically ill patients: a nationwide cohort study in South Korea
Source: Ann Intensive Care. 2023 Jan 13;13:4. doi: 10.1186/s13613-023-01100-5 (PMC9839899; doi:10.1186/s13613-023-01100-5)
Supplement: Supplementary file 1 — Additional file 1: Table S1. Characteristics of three hospital groups (total 369 hospitals). [file 13613_2023_1100_MOESM1_ESM.docx]

Table S1. Characteristics of three hospital groups (total 369 hospitals)

| Variable | | Hospital A  n=72 hospitals | Hospital B  n=293 hospitals | Hospital C  n=4 hospitals |
| --- | --- | --- | --- | --- |
| Type of hospital | |  |  |  |
|  | Tertiary general hospital | 40 (55.6) | 0 (0.0) | 4 (100.0) |
|  | General hospital | 32 (44.4) | 242 (82.6) | 0 (0.0) |
|  | Other hospital | 0 (0.0) | 51 (17.4) | 0 (0.0) |
| Location of hospital | |  |  |  |
|  | Urban area | 41 (56.9) | 116 (39.6) | 4 (100.0) |
|  | Rural area | 31 (43.1) | 177 (60.4) | 0 (0.0) |
| Total number of hospital bed | | 747.2 (181.7) | 256.3 (118.3) | 2,197.8 (413.0) |
| Total number of doctors | | 321.3 (132.0) | 41.0 (41.9) | 1,446.5 (129.9) |
| Total number of nurses | | 758.9 (261.0) | 158.4 (108.6) | 2,688.5 (948.6) |
| Total number of specialist doctor | | 183.3 (70.6) | 33.7 (25.4) | 800.8 (116.0) |
| Total number of pharmacists | | 24.2 (14.5) | 2.8 (3.4) | 123.5 (22.2) |
| Total number of OR | | 15.2 (6.4) | 3.8 (2.5) | 58.3 (14.5) |

OR, operating room
