# Supplementary material for: Trained intensivist coverage and survival outcomes in critically ill patients: a nationwide cohort study in South Korea
Source: Ann Intensive Care. 2023 Jan 13;13:4. doi: 10.1186/s13613-023-01100-5 (PMC9839899; doi:10.1186/s13613-023-01100-5)
Supplement: Supplementary file 2 — Additional file 2: Table S2. Clinicopathological characteristics of all patients (n=1,147,493). [file 13613_2023_1100_MOESM2_ESM.docx]

Table S2. Clinicopathological characteristics of all patients (n=1,147,493)

| Variable | | Mean (SD) or Number (%) |
| --- | --- | --- |
| Age, year | | 68.4 (15.3) |
| Sex, men | | 656,331 (57.2) |
| Having a job | | 597,628 (52.1) |
| Household income level | |  |
|  | Medical aid program | 110,420 (9.6) |
|  | Q1 (Lowest) | 187,247 (16.3) |
|  | Q2 | 169,303 (14.8) |
|  | Q3 | 218,369 (19.0) |
|  | Q4 (Highest) | 336,942 (29.4) |
|  | Unknown | 125,212 (10.9) |
| Residence | |  |
|  | Urban area | 414,297 (36.1) |
|  | Rural area | 622,354 (54.2) |
|  | Unknown | 110,842 (9.7) |
| ICU stay, day | | 3.6 (5.4) |
| LOS, day | | 14.3 (12.5) |
| CCI, point | | 2.5 (2.2) |
| Admitting department | |  |
|  | Non-IM | 563,032 (49.1) |
|  | IM | 584,461 (50.9) |
| Hospital admission through ED | | 664,279 (57.9) |
| Isolated ICU admission | | 32,138 (2.8) |
| Hospital level* | |  |
|  | A | 613,939 (53.5) |
|  | B | 432,242 (37.7) |
|  | C | 101,332 (8.8) |
| Surgery associated hospital admission | | 792,245 (69.0) |
| Mechanical ventilator support | | 288,178 (25.1) |
| ECMO support | | 7,611 (0.7) |
| CRRT use | | 49,940 (4.4) |
| Result of hospitalization | |  |
|  | Same-hospital follow-up | 210,050 (18.3) |
|  | Transfer to a long-term facility care center | 50,949 (4.4) |
|  | Death during hospitalization | 172,801 (15.1) |
|  | Discharge and other outpatient clinic follow-up | 713,693 (62.2) |
| 30 d mortality | | 196,835 (17.2) |
| 90 d mortality | | 262,161 (22.8) |
| 1-year mortality | | 341,414 (29.8) |
| Total cost for hospitalization, USD | | 8,606.9 (8,987.1) |
| Year of admission | |  |
|  | 2016 | 260,470 (22.7) |
|  | 2017 | 274,742 (23.9) |
|  | 2018 | 286,584 (25.0) |
|  | 2019 | 325,697 (28.4) |

*Hospital location, type of hospital, total number of doctors, specialist doctors, nurses, and pharmacists, total number of hospital beds, and total number of operating rooms were used for hierarchical approach to account for clustering at the level of the hospital. Detailed information was presented in eTable 2.

SD, standard deviation; ICU, intensive care unit; LOS, length of hospital stays; CCI, Charlson comorbidity index; IM, internal medicine; ED, emergency department; ECMO, extracorporeal membrane oxygenation; CRRT, continuous renal replacement therapy; USD, United States Dollars
